# The utility of basic blood counts, WBC histogram and C-reactive protein in detecting malaria

**DOI:** 10.1186/s12879-021-06704-5

**Published:** 2021-09-26

**Authors:** Jun Nishimura, Parag Dharap, Sebastien Raimbault

**Affiliations:** 1 Horiba Medical, Kyoto, Japan; 2 Dharap’s Diagnostic Center, Mumbai, India; 3 Horiba Medical, Montpellier, France

**Keywords:** Malaria infection, Hematology analyzer, WBC histogram, CRP

## Abstract

**Background:**

Hematology analyzers display abnormal parameters during malaria infection providing insightful information for suspecting and assessing malaria infection. The goal of this study is to demonstrate the potential of a three-part differential hematology analyzer to assess malaria, provide information about the parasitemia, and discuss the importance of combining C-reactive protein (CRP) with hematology parameters to obtain further information about the malaria infection.

**Methods:**

The present study shows the results of a case–control study during the monsoon season of years 2018 and 2019 in Mumbai, India. The study considers 1008 non-malaria febrile cases, 209 *P. vivax* and 31 *P. falciparum* positive malaria samples, five cases of mixed *P. vivax* and *P. falciparum* infection, and three co-infection cases of *P. vivax* and dengue. Raw data from the three-part analyzer LC-667G CRP (HORIBA) and the corresponding microscopic findings (golden standard for diagnosis of malaria) were obtained for each sample.

**Results:**

The medians of platelet counts (PLT) were 102.5, 109.0, and 223.0 × 10^3^/µL, while CRP medians were 67.4, 81.4 and 10.4 mg/L in *P. vivax*, *P. falciparum* and control groups respectively (p < 0.001 in Mann–Whitney U tests between malaria and control groups). Compared with negative samples, platelets counting less than 161.5 × 10^3^/µL were observed on malaria patients (OR 19.12, 95% CI 11.89–30.75). Especially in *P. vivax* cases, an abnormal peak was frequently observed in the white blood cells (WBC) histogram around the 37fL channel. The events counted around that channel showed a linear correlation with the counting of red blood cells infected predominantly with larger parasitic forms. Parameters like CRP (rs = 0.325, p < 0.001), WBC (rs = 0.285, p < 0.001) and PLT (rs = − 0.303, p < 0.001) were correlated with the parasitemia of *P. vivax* samples. Between the malaria and dengue groups, the highest area under the receiver operating characteristic curve was observed on CRP (0.867, CRP ≥ 26.85 mg/L).

**Conclusions:**

A three-part differential hematology analyzer has the potential to not only trigger malaria diagnosis confirmation but also assess the severity of the infection when CRP is considered.

**Supplementary Information:**

The online version contains supplementary material available at 10.1186/s12879-021-06704-5.

## Background

According to the WHO an estimated 228 million cases of malaria occurred in 2018 worldwide and 85% of this burden was located in 19 sub-Saharan Africa countries and India. For rapid disease management and surveillance of malaria, the WHO recommends two main strategies to eradicate malaria: closing the gaps of healthcare access between urban and rural areas, and diagnosis (before antimalarial treatment) by microscopy or malaria rapid diagnostic tests (RDTs) in all patients with malaria suspicion. Fever is the main manifestation for suspicion of malaria infection. In malaria endemic areas, it acts as a trigger for parasitology testing [1].

RDTs are currently replacing microscopy, the golden standard for diagnosing malaria, especially in endemic countries. Thus, complementary information should be considered to ensure correct interpretation and correct disease management particularly in surveillance programs [2]. The two main limitations of RDTs are the impossibility to obtain parasitemia quantification and, particularly in antibody based RDTs, the persistent positivity after treatment [2]. Microscopy can overcome both limitations by quantifying the parasite density, however the process is highly time-consuming.

## Malaria detection in standard automated cell counters (ACCs)

The potential to diagnose clinically unsuspected cases of malaria for patients subjected to complete blood count (CBC) is one of the main benefits of flagging malaria in hematology analyzers [3]. A comprehensive summary of studies for diagnosing malaria by ACCs without a specific malaria-detection design is well described by Campuzano-Zuluaga [3], in which many of the referred studies provide performances to detect malaria related to abnormalities in the white blood cell (WBC) differentiation channel. ACCs with cytometry capable of differentiating five WBC populations show a clear clustering for malaria population due to specific scattering of infected red blood cells (iRBCs) that are resistant to lyse and/or produce scatters due to residuals of the parasite infection. Pseudoeosinophilia resulting from neutrophils containing haemozoin has been reported as an abnormality caused by malaria infection [4–6]. Fourcade et al. demonstrated that large activated monocytes are present in malaria infections by measuring the standard deviation of the volume of lymphocytes (LYM) and monocytes (MON), calculating a malaria factor with sensitivity of 96.9% and specificity of 82.5% [7]. Many studies have identified a particular cluster by inspecting different angles of light after moderate lysing of red blood cells (RBC) without using extra reagents. However the majority of these studies have a lower performance to detect small or immature forms of parasites due to their low lysing resistance and small size. For example, a recent evaluation on a malaria parameter to evaluate the percentage of iRBCs in the BC-6800 (Mindray) showed a lower sensitivity to detect *P. falciparum* in comparison with detecting *P. vivax* (24.1% and 88.3%, respectively). This is most probably due to the difficulty of detecting early stage parasites and their low nucleic acid content [8], which are prevalent in *P. falciparum* infections where trophozoites and schizonts are barely seen in peripheral blood.

In spite of all promising results from ACCs to identify malaria, fewer studies have been conducted for three-part differential ACCs. One study combined hemoglobin (HGB), total lymphocyte count (TLC), platelets (PLTs) and red cell distribution width (RDW) data in a KX-21 (Sysmex) achieving a 26.1% sensitivity and 96.3% specificity in a study mainly consisting of *P. vivax* infected samples [9]. The majority of small ACCs utilize the Coulter-principle to classify WBCs in populations based on size and obtain an RBC distribution using limited reagents. Identifying intracellular presence of malaria parasites based only on abnormal WBC histogram distributions caused by the infection, poses a difficult challenge.

## C-reactive protein (CRP) to distinguish malaria

CRP is a member of the pentraxin family of proteins, an acute-phase reactant and a biomarker for inflammation. Although CRP is not a specific biomarker for detecting malaria infection, several studies have identified CRP to be a potential marker to assess the severity of malaria infections [10–13]. A study in the south-western part of the Brazilian Amazon has shown evidence in the association of *P. vivax* malaria infection with inflammatory activation, including CRP, and cytokine imbalance [14].

Dengue and malaria infections, which frequently coexist in endemic areas, present with similar symptomatology and produce similar abnormalities in CBC parameters. CRP has been observed to be a useful biomarker to discriminate between malaria and dengue (Epelboin et al.) using CRP > 5 mg/L as a cut off to differentiate malaria from dengue, obtaining sensitivity of 99% and specificity of 35% [15]. In addition, effective management and surveillance of malaria require a screening method facilitating quick transfer of patients to receive diagnostic confirmation and proper treatment. Point-of-care-testing (POCT) hematology analyzers providing information about malaria and other coexisting infections such as dengue could play a major role to support this line of action.

The goal of this study is to demonstrate the potential of a three-part differential hematology analyzer to assess malaria, provide information about the parasitemia, and discuss the importance of combining CRP with hematology parameters to obtain further information about the malaria infection.

## Methods

The present study shows the results of a case–control study involving comparison of raw instrument data from a three-part differential hematology analyzer with CRP measurement (LC-667G CRP, HORIBA) and corresponding microscopic findings of samples obtained from patients visiting Dr. Dharap’s Diagnostic Centre, Mumbai, India during the monsoon season of years 2018 and 2019.

During monsoon season, the laboratory routinely caters to patients for diagnostic testing in cases of acute febrile illness which comprises of a CBC + CRP, peripheral examination of blood smear for malarial parasites and RDTs for malaria and dengue NS1, being an endemic area with coexistent infections. All groups considered for the current study were evaluated as patients with acute febrile illness. Documented historical data of known malaria cases, as well as dengue positive and negative cases were used for the study.

As being the golden standard for diagnosis, malaria positivity was determined solely by the examination of blood smears. We found four discordant cases between the blood smear inspection and the RDTs: one case was confirmed by blood smear but negative in both species RDTs, two samples were positive with *P. vivax* RDTs but negative in blood smear, and one sample was positive with *P. falciparum* RDT but negative in blood smear. For the current study, the historical documented data was further anonymized in coded fashion in order to protect patient confidentiality in accordance with local ethical or IRB guidelines, according to national and international standards for the conduct of clinical studies including 21 CFR Parts 50 and 56 and International Conference on Harmonization (ICH) E6-Good Clinical Practice Consolidated Guideline. Ethical clearance was not obtained as the study was carried as a retrospective review of the available raw instrument and historical patient data, without any collection of extra specimen or monetary charges to patients and did not involve any communication of results of the study to the clinician so as to affect the diagnostic and therapeutic management of the patients.

### Laboratory techniques

Testing for ‘Acute Febrile Illness’ in the laboratory comprises of a full blood count, CRP value, peripheral examination of blood smear for malarial parasites, and RDTs for malaria and dengue NS1.

A full blood count is performed using a three-part differential hematology analyzer (LC-667G CRP, HORIBA) which additionally provides CRP level estimations. For peripheral blood smear examination the following technique is routinely used. Anticoagulated (K2-EDTA) blood of the patient is used to prepare thick & thin blood smears for peripheral blood smear examination (PBSE). Both types of smear preparations are air dried. The thin preparation is fixed with methanol, whereas the thick blood smear is subjected to ‘process of de-hemoglobinisation’ with deionized water. Both smears are then stained by ‘Field staining technique’ using an automated slide stainer. Thin smears are examined for presence of malarial parasites and species identification, presence of platelet aggregates, macroplatelets and nucleated red blood cells, if any.

Malaria cases are further assessed for parasite density by counting parasite life cycle forms against 100 white blood cells by an experienced pathologist. The life cycle forms are grouped as small forms being ring and early ameboids, and large forms including late ameboids with ample parasitic material, schizonts and gametocytes.

For additional screening and confirmation of malaria cases, Rapid Malaria Antigen detection test kits manufactured by SD Biosensor Healthcare Pvt. Ltd., Gurugram, India using monoclonal anti-*P. falciparum* HRP-II (0.75 ± 0.15 µg) with monoclonal anti-*P. vivax* pLDH (0.75 ± 0.15 µg) respectively to detect presence of *P. falciparum* & *P. vivax* related antigens are used.

For screening and diagnosis of dengue, RecombiLISA NS1 Antigen tests are performed on separate serum samples, utilizing pairs of specific polyclonal & monoclonal anti-dengue antibodies of all four serotypes (DEN1, 2, 3, 4) and analytical sensitivity of 0.3 ng/mL for type 2 NS1 antigen, manufactured by CTK Biotech, Inc., United States of America.

### Statistical analysis

To determine the significance of the differences between the hematology parameters extracted from LC-667G CRP of positive & negative samples of *P. vivax* & *P. falciparum* samples respectively, as well as malaria & dengue positive samples, Mann–Whitney U and Krustal–Wallis tests were applied due to their non-parametric distribution.

The Spearman’s rank correlation coefficients were calculated for understanding the independent association of the inspected parameters with the parasites load of *P. vivax* positive samples and to understand if CRP is correlated with the age of patients in a group stratification of malaria species and patient sex. Moreover, to evaluate the hypothesis of CRP values being different between *P. vivax* and *P. falciparum* groups, Mann–Whitney U test was used to evaluate the difference in medians.

A receiver operating characteristic (ROC) curve, the area under the curve (AUC), sensitivity, specificity, and the odds ratios were calculated for evaluating the clinical significance to detect malaria positivity among fever patients and to differentiate malaria from dengue samples.

Due to the confounding factors in all the parameters from the analyzer, particularly CRP, and the limited distribution of samples, a matching method for reducing the risk of selection bias caused by the age and sex of patients was performed only in individual parameter comparisons (i.e., comparison of CRP boxplots between groups, CRP association with clinical variables and the receiver operating characteristic curve). Please refer to the Additional file 1: “Annex 1 QUADAS-2 analysis” for further information about the risk of bias for the present study.

All the whisker-plots and statistical analyses were computed with OriginPro 2021 9.8.0200 and Excel 2016. The histogram graphs were created using R software 3.5.1.

## Results

From the monsoon seasons of 2018 and 2019, based on selection criteria and availability of corresponding data, 209 (144 males, 65 females) *P. vivax* positive cases, 31 (22 males, 9 females) *P. falciparum* samples, five cases of mixed *P. vivax* and *P. falciparum* malaria infection (4 males, 1 female), and three co-infections of *P. vivax* and dengue (2 males, 1 female) were considered for the present study. Nonetheless, the mixed malarial infections, and the three *P. vivax* and dengue co-infection samples were excluded from all statistical tests. In addition, 1008 non-malaria febrile cases were examined including 197 (124 males, 73 females) dengue positive cases. The median and range of ages were 28 (1–91), 35 (5–87) and 36 years (15–77) for non-malarial, *P. vivax* and *P. falciparum* cases, respectively.

Using the Krustal–Wallis test to compare the four groups (see Table 1), median values of WBCs, PLTs, plateletcrit (PCT) and %LYM were lower in malaria and dengue samples compared to negative ones with a statistical difference (p < 0.05). Contrary, the median values of mean corpuscular hemoglobin concentration (MCHC), mean platelet volume (MPV), platelet distribution width (PDW), and %MON were higher than negative samples. RBC, HGB, hematocrit (HCT), mean corpuscular volume (MCV), mean corpuscular hemoglobin (MCH) and granulocytes percentage (%GRA) showed no significant difference in median between the four groups.

**Table 1:** Hematology parameters statistics of the LC-667G of data sets 2018 and 2019

	*P. vivax* (n = 209) Median (IQR)	*P. falciparum* (31) Median (IQR)	Dengue (n = 197) Median (IQR)	Patients with fever but Non-malaria non-dengue (n = 811) Median (IQR)	*p*_*KW*_	*p*_*VF*_	*p*_*VD*_	*p*_*FD*_	*p*_*VN*_	*p*_*FN*_
WBC (10^3^/µL)	5.4 (4.1–6.6)	4.7 (3.8–5.5)	4.4 (3.4–6)	6.6 (5.1–8.5)	< 0.001	0.056	< 0.001	0.404	< 0.001	< 0.001
RBC (10^6^/µL)	4.67 (4.3–5.15)	4.5 (4.09–4.89)	4.74 (4.4–5.18)	4.73 (4.36–5.09)	0.19	0.255	0.437	0.199	0.509	0.119
HGB (g/dL)	13.5 (12.32–14.67)	12.7 (11.8–14.4)	13.4 (12.5–14.6)	13.3 (11.95–14.8)	0.24	0.082	0.894	0.114	0.203	0.206
HCT (%)	40.05 (36.32–43.4)	37.7 (35.8–42.4)	40.3 (37.3–43.5)	39.7 (35.72–43.87)	0.318	0.136	0.436	0.053	0.601	0.187
MCV (µm^3^)	86.05 (82–89.37)	84.2 (74.6–89.9)	84.8 (81.1–89)	85.4 (80.6–89.8)	0.437	0.148	0.667	0.309	0.358	0.335
MCH (pg)	29.15 (27.5–30.47)	28.7 (25.9–30.9)	28.6 (27.1–30.1)	28.6 (26.75–30.3)	0.123	0.185	0.139	0.669	0.04	0.593
MCHC (g/dL)	33.80 (33.2–34.75)	33.80 (33–34.4)	33.35 (33.1–33.9)	33.50 (32.9–33.9)	< 0.001	0.461	< 0.001	0.115	< 0.001	0.238
RDW (%)	14.45 (13.8–15.1)	15 (13.6–15.6)	14.5 (14.1–15.2)	14.7 (14.2–15.4)	< 0.001	0.333	0.152	0.09	< 0.001	0.698
PLT (10^3^/µL)	102.5 (66.25–149)	109 (79–140)	174 (136–219)	223 (174–274)	< 0.001	0.991	< 0.001	< 0.001	< 0.001	< 0.001
MPV (μm^3^)	8.4 (7.9–9)	8.6 (7.8–9.3)	8.2 (7.7–8.8)	8.1 (7.6–8.6)	< 0.001	0.168	0.732	0.14	< 0.001	0.001
PCT (%)	0.0865 (0.056–0.119)	0.088 (0.063–0.113)	0.144 (0.118–0.175)	0.179 (0.143–0.217)	< 0.001	0.769	< 0.001	< 0.001	< 0.001	< 0.001
PDW (%)	15.35 (13.9–17.3)	16.9 (13.8–19.4)	15 (14–16.4)	14.6 (13.7–15.9)	< 0.001	0.274	0.122	0.102	< 0.001	0.023
%LYM	25.9 (20.4–34.2)	22.8 (18.5–27)	23.4 (16.75–31.22)	26.1 (18–35.3)	0.016	0.029	0.015	0.919	0.406	0.117
%MON	5.6 (4–7.3)	5.8 (4.4–7.2)	5.5 (4.2–7.2)	5.2 (4–6.8)	0.04	0.658	0.217	0.636	0.055	0.156
%GRA	68 (59.1–73.8)	71.2 (66.3–77.2)	70.35 (61.55–79.25)	68.4 (58.32–77.6)	0.071	0.061	0.053	0.876	0.257	0.236
CRP (mg/L)	67.4 (35.7–111.2)	81.4 (37.5–122.3)	9.7 (3.3–27.1)	10.4 (2.9–27.9)	< 0.001	0.455	< 0.001	< 0.001	< 0.001	< 0.001

p-value of less than 0.05 was considered statistically significant

${\mathrm{p}}_{\mathrm{K}\mathrm{W}}$, ${\mathrm{p}}_{\mathrm{V}\mathrm{F}}$, ${\mathrm{p}}_{\mathrm{V}\mathrm{D}}$, ${\mathrm{p}}_{\mathrm{F}\mathrm{D}}$, ${\mathrm{p}}_{\mathrm{V}\mathrm{N}}$, and ${\mathrm{p}}_{\mathrm{F}\mathrm{N}}$ corresponds to the p-value of Kruskal–Wallis test, PV vs PF, PV vs dengue, PF vs dengue, PV vs negative, and PF vs negative, respectively

The medians of the parameters that had a significant difference between *P. vivax* infected and negative samples were WBCs, MCH, MCHC, RDW, PLTs, MPV, PCT, PDW and CRP. Similarly, *P. falciparum* cases had the same parameters with significant differences except in MCH, MCHC and RDW.

**Fig. 1:**
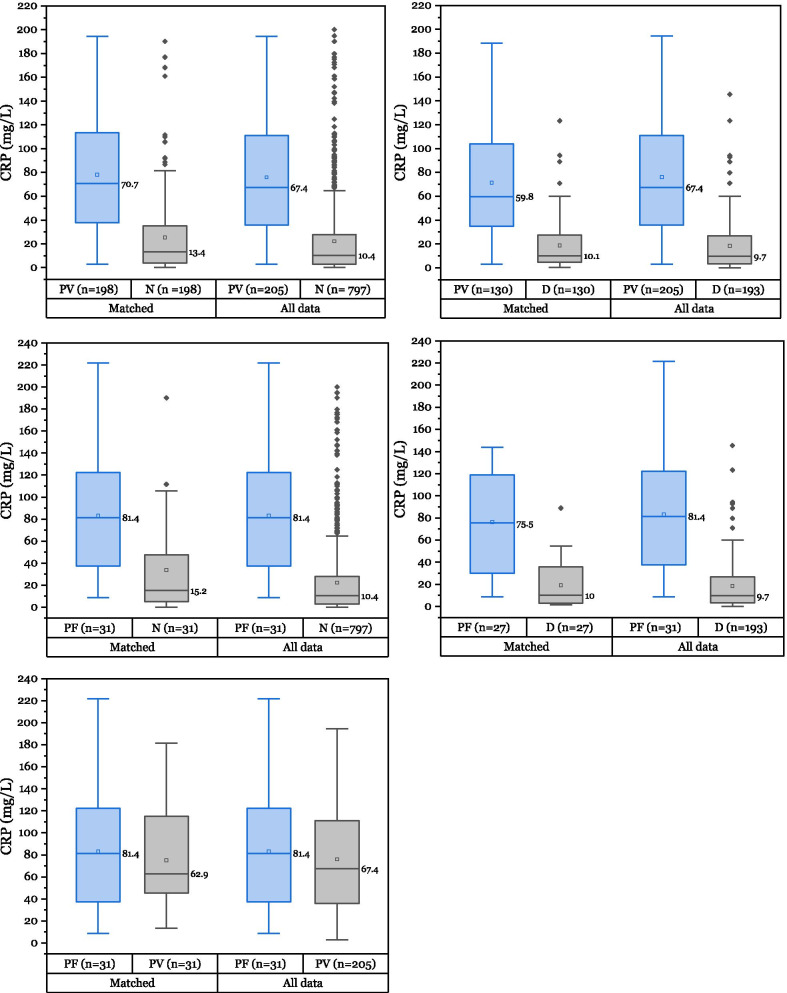
Comparison of CRP distribution among different groups. *P. vivax* (PV), *P. falciparum* (PF), dengue (D) and non-malaria-dengue samples (N) distributions are represented with their corresponding median value and outliers. All comparisons left box-plots were matched in age (± 2 years) and sex

Comparing both malaria infected cases with dengue, only PLT, PCT and CRP medians had a significant difference, which makes it difficult to differentiate between both diseases when observing solely the hematology parameters affection. The greatest difference of median between malaria and dengue infected patients was observed with CRP (Fig. 1).

### Parasitemia association with different hematology parameters

To inspect the affection of the hematology parameters regarding the parasite density observed in peripheral blood, only the data-set of *P. vivax* samples in the current study had enough data variation to provide interesting results. The distribution of parasitemia in *P. falciparum* samples of the present study is limited, more than 60% of the cases had less than 0.2% of parasitemia, thus the analysis of associating parasitemia with the impact on hematology parameters was performed solely for the *P. vivax* set of data.

The parasitemia percentage establishes the relation of the number of parasites found in thin smears against the total count of RBCs and was calculated with the following formula:

1 $$\% {\text{Parasitemia}} = \frac{{\frac{{\# Parasites}}{{100\,WBC}}\left( {\frac{{\# WBC}}{{\mu L}}} \right)}}{{\frac{{\# RBC}}{{\mu L}}}}~ \times 100$$

The total counts of WBCs and RBCs were retrieved from the hematology analyzer.

The parasitemia ranged from 0.00005 to 2.15% and no cases of high parasitemia (> 5%) were found. Table 2 describes the Spearman’s rank correlation coefficients of the resulting parameters by the LC-667G in relation with the total parasitemia percentage of *P. vivax* positive samples. The parameters with statistically significant positive correlations with the total parasitemia percentage were WBC, RDW, PDW, %LYM and CRP. Whereas negative correlations with statistical significance were observed in HGB, PLT, PCT and %MON.

**Table 2:** Spearman’s rank correlation coefficients and their corresponding p-value of the inspected parameters against the total parasitemia percentage of *P. vivax* samples

	%Parasitemia r_s_	p-value
WBC	0.285	< 0.001
RBC	− 0.128	0.068
Hgb	− 0.141	0.045
Hct	− 0.130	0.064
MCV	− 0.026	0.705
MCH	− 0.037	0.598
MCHC	− 0.037	0.594
RDW	0.138	0.049
PLT	− 0.303	< 0.001
MPV	0.078	0.265
PCT	− 0.283	< 0.001
PDW	0.179	0.010
%LYM	0.143	0.042
%MON	− 0.338	< 0.001
%GRA	− 0.057	0.416
CRP	0.325	< 0.001

10 samples from the original data set were incomplete on parasites counting, therefore they were not contemplated for the calculation of Table 2 results

p-value of less than 0.05 was considered statistically significant

The CRP measurement value has many confounding factors including patient’s age and sex. In order to evaluate the association and possible confounding factors of the available clinical variables with CRP value, a stratification of groups was created by matching patient age and sex between positive groups of *P. falciparum* (n = 31, mean age = 39.35, SD = 17.33) and *P. vivax* (n = 31, mean age = 39.54, SD = 17.66). Table 3 shows the comparison of CRP medians between matched groups of *P. vivax* and *P. falciparum*, and each patient sex group. In addition, the Spearman’s rank correlation coefficients between age and CRP value in each subgroup of malaria species were calculated. The null hypotheses of equality of medians were not rejected when comparing the CRP values between malaria species (p-value = 0.526), nor in patient sex of each malaria specie group. CRP correlates positively with patient age in the *P. falciparum* group (p = 0.008), but not in the *P. vivax* group (p = 0.825).

**Table 3:** CRP value association with malaria species, patient sex and age

CRP (mg/L)					
*P. vivax* (31)			*P. falciparum* (31)		p-value
Md = 62.9			Md = 81.4		0.526
Age correlation			Age correlation		
rs = 0.041, p = 0.825			rs = 0.465, p = 0.008		
Male (22)	Female (9)	p-value	Male (22)	Female (9)	p-value
Md = 67.45	Md = 50.4	0.727	Md = 75.15	Md = 88.6	0.372

A matching method of patient sex and age between positive *P. vivax* and *P. falciparum* groups was conducted

p-value of less than 0.05 was considered statistically significant

### WBC histogram interference in the RBC ghosting area

**Fig. 2:**
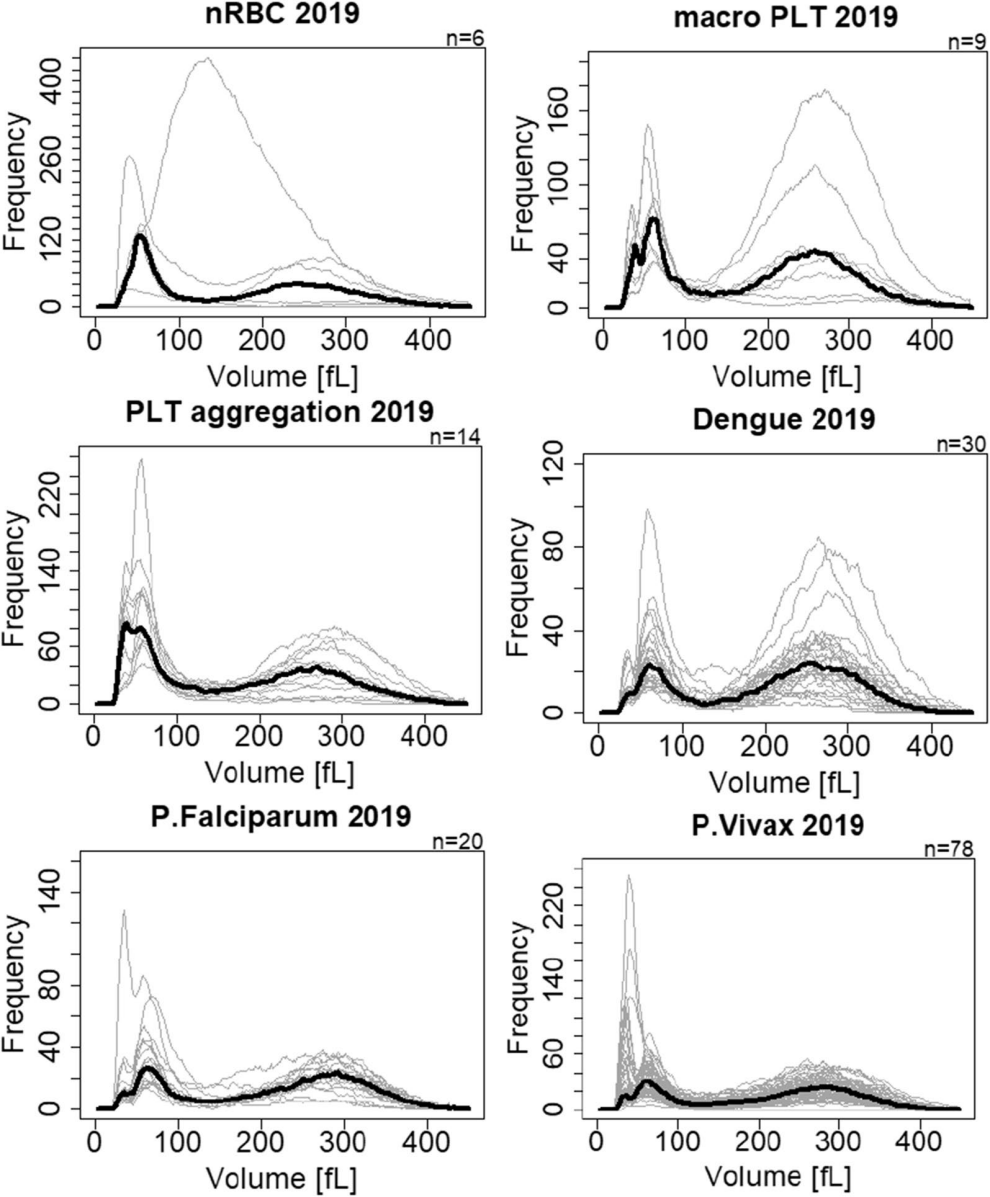
WBC histograms of 2019 data-set with interferences around 37-fL. Bold lines represent the median of the histograms

**Fig. 3:**
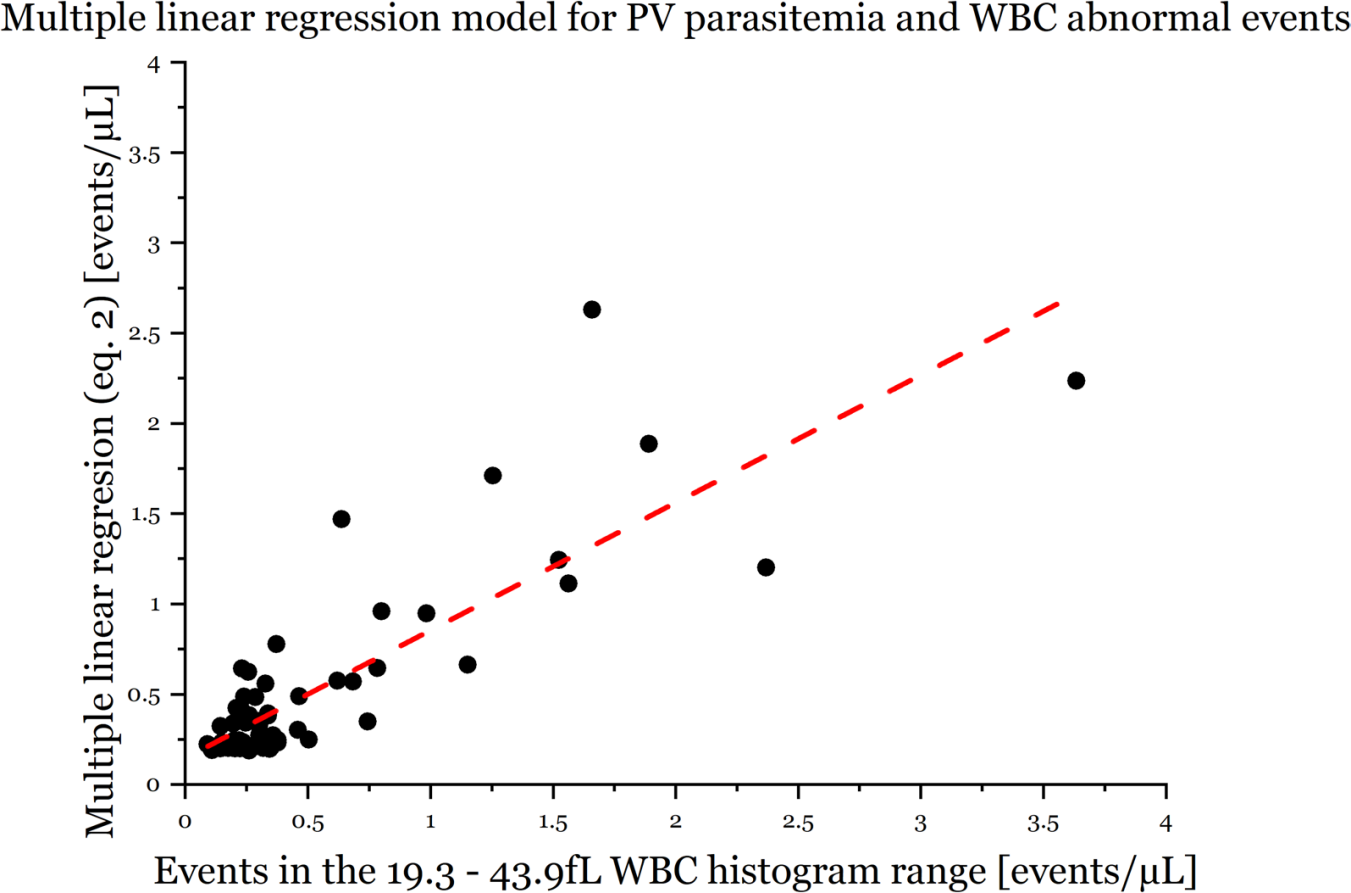
Multiple linear regression model between the events before 43.9fL and parasite density. The vertical axis corresponds to the number of events from 19.3fL to 43.9fL in the WBC histogram and the horizontal axis to the parasite density of small and large forms of samples infected by malaria *P. vivax*. The regression model shows that the contribution of the linearity is mainly provided by large forms of *P. vivax* malaria infection

In the LC-667G CRP HORIBA analyzer, an abnormal peak located approximately at the 37fL channel in the WBC histogram appears mainly in malaria *P. vivax* samples with high density of large forms of parasites (see Fig. 2). The classification of parasite forms in the data set of 2018 was not available. Therefore only the parasite density information from dataset 2019 of *P. vivax* samples were used (n = 68, 10 samples were excluded due to incomplete information). A multiple linear regression model was calculated obtaining the following equation $y=0.184+2.238\times {10}^{-5}{\alpha }_{1}+6.007\times {10}^{-5}{\alpha }_{2}$, where *y* represents the events per µL from 19.3fL to 43.9fL in the WBC histogram (refer Eq. 2), and ${\alpha }_{1}$ and ${\alpha }_{2}$ corresponds to the small and big parasite forms per µL identified in thin smears by microscopy, respectively. The contribution of large forms is approximately 2.7 times bigger than the contribution of small forms $({{\alpha }_{2}/\alpha }_{1})$ and the R-squared of the linear model is equal to 0.707 (see Fig. 3). Additionally, from the correlation matrix, the small and large forms of parasitemia showed a correlation of − 0.315.

2 $$Events\,per\,\mu L\,from\,~19.3{\text{f}}\,{\text{L}}\,{\text{to}}\,{\text{~}}43.9\,{\text{fL}} = \frac{{{\text{Sum~}}\,{\text{events~}}\,{\text{19}}{\text{.3}}\,{\text{fL~to~43}}{\text{.9}}\,{\text{fL}}}}{{Total\,events\,of\,WBC\,~hist}} \times \left( {\# WBC \times 10^{3} } \right)~\,\left[ {\frac{{events}}{{\mu L}}} \right]$$

Large forms of parasites in *P. falciparum* samples are barely seen in peripheral blood due to sequestration in the spleen, thus interference in the RBC ghosting area of the WBC histogram will be smaller than in *P. vivax* samples where large forms of parasites remain in peripheral blood (compare subplots of Fig. 2). In the analyzed set of data, different interferences not-related to parasite inclusion located in the 37-fL area are displayed in Fig. 2. These interferences include platelet aggregations, macro platelets, and nRBCs.

### Analysis on the association and performance of hematology parameters and CRP to identify malaria

**Fig. 4:**
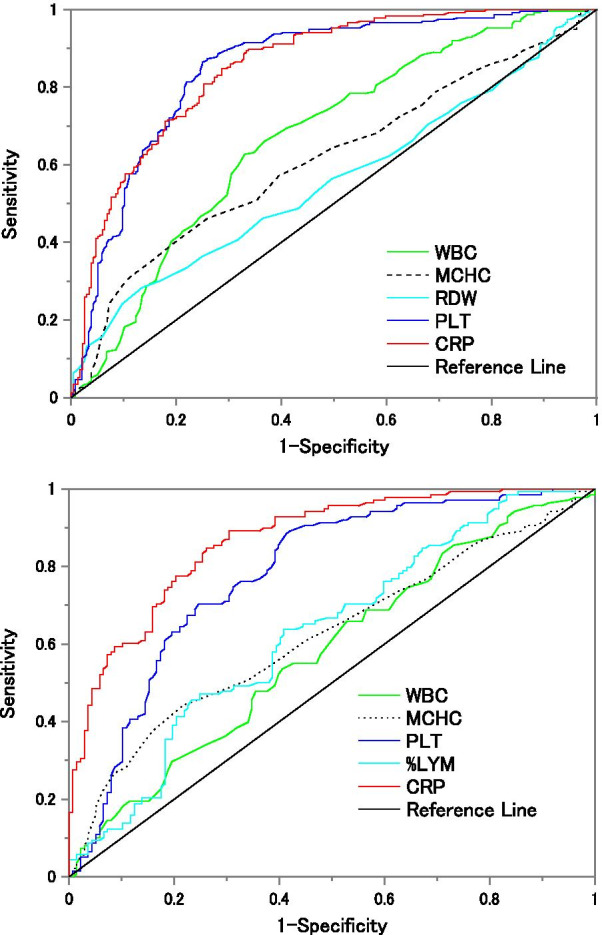
Upper graph: ROC curve of all malaria positives against negative samples. Lower graph: ROC curve of all malaria positives against dengue samples. Compared groups were matched in age (± 2 years) and sex, 236 and 138 matched samples for negative and dengue groups, respectively

From the statistically significant medians between malaria positive samples and the remaining samples (refer to Table 1), excluding derived parameters like PCT and PDW, calculations of the receiver operating characteristic (ROC) curve were included in the study to understand the clinical relevance of each abnormal parameter to predict malaria infection. Figure 4 shows the ROC curves that analyze matched malaria positive samples against negative and dengue samples. The AUC of the inspected parameters is displayed in Table 4 along with the sensitivity, specificity, and odds ratio for the cut-off value using the Youden index.

**Table 4:** AUC, Youden’s index and accuracy results of single parameters with statistical significance between medians of malaria positive, malaria negative, and dengue positive samples

Parameter	Malaria vs Negative samples				Malaria vs dengue samples			
	AUC	Cut-off value at YI	Sens.% Spe. %	Odds ratio (CI 95%)	AUC	Cut-off value at YI	Sens.% Spe. %	Odds ratio (CI 95%)
WBC	0.672	≤ 5.75 (10^3^/µL)	62.71% 66.94%	3.40 (2.33–4.97)	0.580	≥ 4.35 (${10}^{3}$/µL)	65.94% 47.10%	1.72 (1.06–2.80)
MCHC	0.613	≥ 33.95 (g/dL)	39.83% 80.42%	2.71 (1.79–4.11)	0.620	≥ 33.85 (g/dL)	44.20% 78.26%	2.85 (1.68–4.82)
RDW	0.558	≤ 13.85 (%)	28.39% 86.44%	2.52 (1.58–4.03)				
PLT	0.849	≤ 161.5 (10^3^/µL)	86.44% 75.00%	19.12 (11.89–30.75)	0.780	≤ 160.5 (10^3^/µL)	88.40% 58.69%	10.83 (5.81–20.17)
%LYM					0.628	≥ 24.6 (%)	63.76% 59.12%	2.54 (1.56–4.14)
CRP	0.852	≥ 27.65 (mg/L)	84.74% 71.36%	13.84 (8.79–21.80)	0.867	≥ 26.85 (mg/L)	84.78% 73.91%	15.78 (8.66–28.76)

The sensitivity, specificity, and odds ratios were calculated with the cut-off values of the Youden’s index. Negative samples were matched with malaria positive samples in age and sex

## Discussion

The most commonly reported affections on hematology parameters observed in malaria infection are thrombocytopenia, anemia, and leucopenia [16, 17]. Thrombocytopenia in malaria infections occurs due to complex platelet roles and abnormalities, including enabling cytoadhesion of iRBCs to endothelial cells, clumping and killing iRBCs [18], coagulation disturbances, bone marrow alterations, antibody-mediated platelet destruction, systemic platelet activation, vascular pooling, and oxidative stress [19]. Moreover, in hematological analyzers, agglutination of PLTs would lead to an apparent platelet loss due to shortcomings of the impedance method [20]. In the present study, thrombocytopenia (< 150 ${10}^{3}$/µL) was predominantly observed in malaria *P. falciparum* cases (83.87%), rather than *P. vivax* (76.44%), which is similar to the observations reported in [21, 22]. Authors in [20] have concluded that in malaria cases, in addition to type of species, the severity of thrombocytopenia is also affected by parasite density. Furthermore, supporting this observation, our data indicates a negative correlation in *P. vivax* parasite density with PLT (p < 0.001). To analyze the thrombocytopenia dependency on malaria species, the analysis must considers the parasite load and patient condition, as pointed out in a study where parasitemia was significantly higher in individuals with thrombocytopenia only in *P. vivax*, but not in *P. falciparum* infections [23].

Among the hematology parameters with significant statistical difference in medians between malaria positive and negative samples, platelets related parameters, especially MPV, should be further analyzed due to the strong affection with the elapsed time between collection and measurement of sample.

### Interference in the WBC channel and association with parasitemia

In both three and five part differential ACCs a peak around the RBC ghosting area in the WBC histogram has been identified as one of the main abnormalities to predict malaria infection [24–29]. In the present study, the number of events within this abnormal peak were correlated with the parasite density of *P. vivax* large forms, supporting the same observation of moderate correlation (r = 0.707) in [28]. The abnormal peak under malaria infection is caused mainly by the lysing-resistant large forms of parasites, regardless of the species, and is probably affected by the presence of PLT aggregation and/or macro PLTs. The abnormal peak is less often observed in *P. falciparum* infections as the larger forms are sequestered by the spleen. Mature trophozoites and schizonts in *P. falciparum* infections are barely present in peripheral blood samples. According to [30], the presence of schizontaemia, especially in *P. falciparum* infections, can be used as a marker for disease severity. Therefore a semi-quantitative estimation of schizontaemia can be inferred from the events located before the 37fL channel of the LC-667G WBC histogram and might help to assess the severity of malaria infections.

The abnormal peak in the WBC histogram is not exclusively due to malaria iRBCs. Common interferences include RBC lysed debris, PLT aggregations, macro-PLTs, nRBCs, and others. Researchers used this abnormal peak along with two other parameters of the LH780 (Beckman Coulter) to identify malaria infections having an 83.1% specificity but failed to discriminate from samples with nRBCs [28]. The distribution pattern of the abnormal peak might provide a way to differentiate the exact cause of it. It was observed that PLT aggregations or macro PLTs show a bigger/wider distribution due to the random nature of formation, whereas gametocytes, schizonts and late stages of trophozoites show a narrower size distribution having a smaller dispersion close to the abnormal peak area.

Moreover, considering the parameters that are correlated with the total percentage of parasitemia such as WBC, PLT, and CRP, it might be possible to assess the level of parasitemia in combination with the WBC histogram analysis, especially in *P. vivax* infections. The positive and negative correlations of the *P. vivax* parasitemia with %LYM (p = 0.042) and %MON (p < 0.001) respectively, could be related to the interference in the WBC histogram, which suggests an incorrect counting of WBC subpopulations when iRBCs are present.

### A parameter to increase the specificity of detecting malaria: CRP

In low cost ACCs where the Coulter principle is the only technology responsible for differentiating the WBC population into three parts based on volume, the identification of malaria or dengue infected samples is rather difficult due to the impossibility of differentiating granulocytes populations. Addition of CRP increases the specificity of detecting malaria due to the inflammatory response secondary to parasite infection.

Supporting observations of [31–35], in this study no significant difference of CRP value was observed between medians of matched *P. vivax* and *P. falciparum* groups (p = 0.526)*.* The non-statistical significance in age and CRP correlation in the *P. vivax* group might be affected by the wider distribution of parasite density (cf. *P. falciparum* group). The parasitemia was not included in the current study’s matching method due to the limited variation on P. falciparum positive samples. However, for future analyses on CRP, parasitemia should be also considered and treated as a confounding variable.

Dengue and malaria in many countries are co-endemic diseases that present similar clinical presentations. The importance of distinguishing them has been reported by [36], who developed a tree decision model for discriminating malaria and dengue infections. Dengue samples collected in the present study had a lower count of WBCs and MCHC in comparison to both malaria species supporting observations of [36]. Only for *P. vivax* samples, the difference between medians was statistically significant. Nonetheless, both variables have a poor clinical relevance for differentiating malaria from dengue samples using the Youden’s index (AUC of WBC = 0.580 and AUC of MCHC 0.620). In contrast, PLT medians of both species of malaria infections were lower than dengue cases (p < 0.001) and the odds ratio of malaria positive patients having less than 160 × 10^3^ platelets/μL was 10.83. Discrepancy of results with [36] might be related to the severity of each disease. For example, the hematology parameters affection in dengue infections is greatly dependent on the day of fever. Unfortunately, current data does not include any information about the fever progression of dengue infected individuals.

The CRP parameter showed the most significant difference between malaria and dengue samples. From our current data set, patients with malaria are 15.78 times more likely to present CRP greater than 26.85 mg/L, compared to dengue patients. In comparison with negative patients with fever, patients with malaria have a smaller odds ratio (OR 13.84, CI 8.70–21.80) to present CRP greater than 27.65 mg/L. Although CRP is not a specific biomarker for malaria, the response of CRP to virus infections is rather small, making it a good parameter to understand the etiology of the infection and to help differentiate malaria and dengue from similar hematology parameters. Nevertheless, dengue and other non-viral infections can co-exist in a single sample, thus relying only on CRP should not be an exclusive criteria in differentiating both diseases.

In general, hematology parameters and CRP depend on clinical characteristics and might vary upon demographics. Additionally, the immune response against malaria, dengue or other infections is related to the condition of the patient and the disease’s stage. Caution to generalize the current results must be taken considering the limited distribution of parasites in the inspected samples (particularly in *P. falciparum*), the single location of the study, and the bias selection from retrospective results.

## Conclusions

The LC-667G CRP, a three-part differential hematology analyzer has the potential to not only trigger malaria diagnosis confirmation, but also to assess the severity of the infection due to simultaneous CRP estimations. The current study suggests that CRP could be helpful for distinguishing malaria from dengue. In addition to CRP, the abnormal peak located around the 37fL channel in the WBC histogram can provide a good estimation of the number of large parasites present during the infection. However the calculation might be interfered by other particles such as nRBCs and PLT aggregations. The combination of providing hematology parameters plus CRP values in a hematology analyzer makes a preferred method for screening malaria on a routine basis in cases of any infection suspicion. However, the sole inspection of these abnormal parameters should not by any means replace the use of the golden standard methods for diagnosing malaria (i.e., microscopy inspection and RDTs).

Our current data set is limited by the number of *P. falciparum* samples. The discussion and observations provided were focused on *P. vivax* positive cases. Further research following this study should be oriented to measure *P. falciparum* samples, to evaluate the potential of using hematology analyzers to provide additional information to the RDTs, and to monitor anti-malaria drugs treatments.

## Supplementary Information

**Additional file 1: Annex 1.** Annex 1 QUADAS-2 analysis.

**Additional file 2: Annex 2.** Raw data.

## Data Availability

The datasets used and/or analyzed during the current study are available in the Additional file 2: “Annex 2 Raw data”. The age and gender information was removed to protect patient anonymity.

## Abbreviations

WHO: World Health Organization
RDT: Rapid diagnostic test
ACC: Automated cell counter
CBC: Complete blood count
WBC: White blood cell
LYM: Lymphocytes
MON: Monocytes
RBC: Red blood cell
iRBC: Infected red blood cell
HGB: Hemoglobin
TLC: Total lymphocyte count
RDW: Red cell distribution width
CRP: C-reactive protein
POCT: Point of care testing
PLT: Platelet
PCT: Plateletcrit
MCHC: Mean corpuscular hemoglobin concentration
MPV: Mean platelet volume
PDW: Platelet distribution width
HCT: Hematocrit
MCV: Mean corpuscular volume
MCH: Mean corpuscular hemoglobin
GRA: Granulocytes
CV: Coefficient of variation
nRBC: Nucleic red blood cell
