# Supplementary material for: The utility of basic blood counts, WBC histogram and C-reactive protein in detecting malaria
Source: BMC Infect Dis. 2021 Sep 26;21:1006. doi: 10.1186/s12879-021-06704-5 (PMC8474782; doi:10.1186/s12879-021-06704-5)
Supplement: Supplementary file 1 — Additional file 1: Annex 1. Annex 1 QUADAS-2 analysis. [file 12879_2021_6704_MOESM1_ESM.docx]

**Annex 1. QUADAS-2 analysis**

Domain 1. Patient selection

Risk of bias: Could the selection of patients have introduced bias?

Signalling question 1: Was a consecutive or random sample of patients enrolled?

Yes. Eligible patients were selected from historical data of all patients visited Dr. Dharap Diagnostic Centre during 2018 and 2019 monsoon season. Within these periods all consecutive patients with fever were included in the study. We considered the control group to be patients with fever to include a similar condition to the positive group (malaria diagnosis is triggered by fever). The patients of this group came from the same population.

Signalling question 2: Was a case-control design avoided?

No. We retrospectively selected the patients with only fever manifestations to perform the index tests and reference standards (microscopy smearing for malaria and RDT dengue NS1). All these patients were included in the analysis and receive the same reference standard testing. However non-febrile patients and patients who visited the laboratory outside the monsoon period were excluded.

Signalling question 3: Did the study avoid inappropriate exclusions?

No. Some data was excluded due to incorrect measurements of the hematology analyzer and no replica was taken due to the retrospective nature of the study.

Domain 2. Index test

Risk of Bias: Could the conduct or interpretation of the index test have introduced bias?

Signalling question 1: Were the index test results interpreted without knowledge of the results of the reference standard?

Yes. The CBC+CRP, malaria smearing and dengue NS1 RDTs are conducted by a different technician without interference of each result within four hours of sample extraction. Interpretation of results do not interfere with the reference standard.

Signalling question 2: If a threshold was used, was it pre-specified?

No. The threshold was selected by the Youden Index of the current results’ ROC curve.

Domain 3. Reference standard

Risk of Bias: Could the reference standard, its conduct, or its interpretation have introduced bias?

Signalling question 1: Is the reference standard likely to correctly classify the target condition?

Yes. The golden standard for diagnosing malaria is by identification of parasite in peripheral blood smears.

Signalling question 2: Were the reference standard results interpreted without knowledge of the results of the index test?

Yes. The CBC+CRP, malaria smearing and dengue NS1 RDTs are conducted by a different technician without interference of each result within four hours of sample extraction. Interpretation of results do not interfere with the reference standard.

Domain 4.

Risk of Bias: Could the patient flow have introduced bias?

Signalling question 1: Was there an appropriate interval between index test and reference standard?

Yes. The CBC+CRP, malaria smearing and dengue NS1 RDT are conducted by a different technician without interference of each result in the within three hours of sample extraction. Interpretation of results do not interfere with the index test.

Signalling question 2: Did all patients receive the same reference standard?

Yes. All selected samples from the recorded data received the same reference standard and index tests (the internal protocol at Dr. Dharap Diagnostic Centre for fever patients during monsoon season is to conduct CBC+CRP, malaria smearing, and RDT Dengue NS1 & malaria).

Signalling question 3: Were all patients included in the analysis?

Yes. All patients with the criteria of having fever during monsoon season of 2018 and 2019 were included

**Flowchart**

**
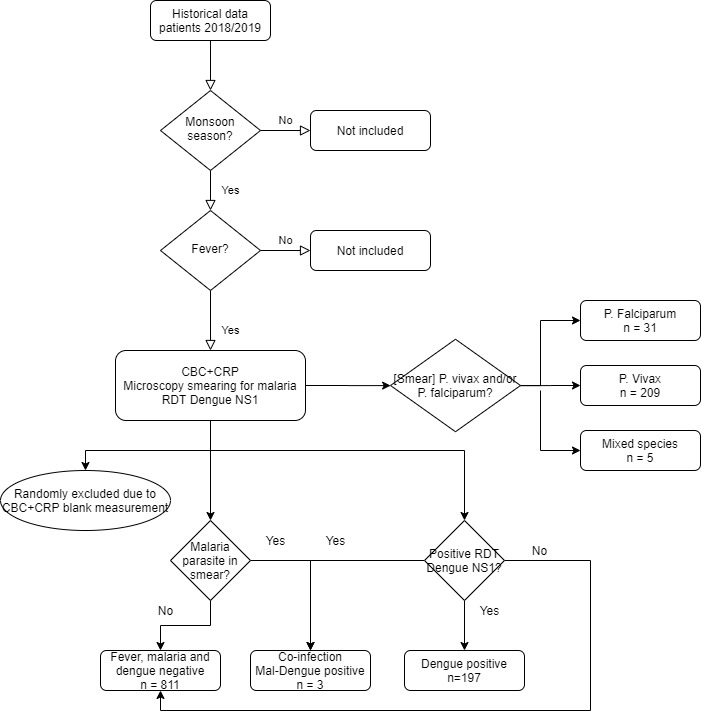
**

**Judgement on bias and applicability**

|  | Risk of bias | | | | Applicability | | |
| --- | --- | --- | --- | --- | --- | --- | --- |
|  | Patient selection | Index test | Reference Standard | Flow and timing | Patient selection | Index test | Reference Standard |
| Study | High risk | High risk | Low risk | Low risk | High risk | High risk | Low risk |

Applicability

Patient selection. Are there concerns that the included patients and setting do not match the review question? The review question is to identify the abnormal parameters coming from a 3DIFF+CRP machine to assess malaria infection. The demographic features of the current study are limited to Mumbai India in a malaria P. vivax cluster during monsoon seasons.

Index test. Are there concerns that the index test, its conduct, or interpretation differ from the review question? The threshold selected to evaluate CRP for diagnosis of malaria was not pre-specified.

Reference standard. Are there concerns that the reference standard, its conduct, or interpretation differ from the review question? The smear inspection was performed on thin and thick slides of peripheral blood collection. The detection of at least one malaria parasite indicates positivity of malaria infection.
